# LINC00958 may be a new prognostic biomarker in various cancers: A meta-analysis and bioinformatics analysis

**DOI:** 10.3389/fgene.2022.998442

**Published:** 2022-11-11

**Authors:** Helin Zhang, Guangming Zhang, Fan Zhang, Xiaochun Yang, Erqiang Li, Bo Wang, Peng Xu, Dengxiao Zhang, Lijun Guo, Xiande Huang

**Affiliations:** ^1^ The First Clinical Medical College of Gansu University of Chinese Medicine (Gansu Provincial Hospital), Lanzhou, China; ^2^ Gansu Provincial Hospital, Lanzhou, China

**Keywords:** bioinformatics analysis, cancers, LINC00958, lncRNA, meta-analysis, prognosis

## Abstract

**Background:** There have been many studies on long non-coding RNAs (lncRNAs) as tumor markers. LINC00958 is a lncRNA that has been studied in a variety of tumor types. This meta-analysis aims to explore the relationship between LINC00958 and clinical prognosis and pathological characteristics in various cancers.

**Methods:** We searched for related studies from PubMed, Web of Science, The Cochrane Library and Embase (up to October 2021). The association of LINC00958 expression with clinicopathological characteristics and prognosis was evaluated using the pooled odds ratios (ORs) or hazard ratios (HRs) with 95% confidence intervals (CIs).

**Results:** 16 studies (1,121 patients) were included in this meta-analysis, we found that overexpression of LINC00958 was associated with poor overall survival (OS) (HR = 1.84; 95% CI: 1.36–2.49; *p* < 0.001). We also found that LINC00958 overexpression was correlated with positive lymph node metastasis (LNM) (OR = 1.91; 95% CI: 1.39–2.63; *p* < 0.001), advanced degree of infiltration (OR = 1.64; 95% CI: 1.11–2.41; *p* = 0.013), advanced tumor-node-metastasis (TNM) stage (OR = 2.80; 95% CI: 1.48–5.33; *p* = 0.002). Other clinicopathological characteristics have no obvious correlation, such as age, sex, tumor size, distant metastasis, and differentiation grade (*p* > 0.05).

**Conclusion:** In summary, the overexpression of LINC00958 is significantly correlated with poor OS, positive LNM, advanced degree of infiltration, and advanced TNM stage. LINC00958 might serve as a potential prognostic biomarker and therapeutic target for a variety of cancers. However, rigorous studies with large sample sizes are still needed for further research and demonstration.

## Introduction

In 2020, there were approximately 19.3 million new cancer cases and 10 million cancer deaths worldwide (Sung et al., 2021). Due to the impact of COVID-19, there will be a significant reduction in cancer diagnosis and treatment, followed by an increase in advanced cancer detection and death rates (Siegel et al., 2021). Despite the variety of treatments available today, the recurrence and mortality rates of cancer are still high, leading to increased costs and poor prognosis. Accurate early diagnosis and prognosis prediction are crucial for improving (Huang et al., 2020). According to existing researches, biomarkers are helpful for early diagnosis and prognosis prediction of tumors (Dunn et al., 2010). Long non-coding RNAs (lncRNAs) have been studied for use as biomarkers and have been found to be involved in many illnesses (Renganathan and Felley-Bosco, 2017).

LncRNAs were initially understudied and poorly understood. In the last few years, numerous studies have identified lncRNAs associated with many diseases, especially cancer (Renganathan and Felley-Bosco, 2017). Therefore, lncRNA may be used as biomarkers to detect cancer occurrence and predict cancer prognosis. Mutations and disorders of lncRNA may be closely related to diseases, especially affecting the development and differentiation of cells (Kazimierczyk et al., 2020). The current research has found that lncRNA is involved in gene expression regulation, involving multiple mechanisms such as selective splicing, etc (Gibb et al., 2011; Yang et al., 2014; Yang et al., 2020b).

LINC00958 is one of these lncRNAs found to be associated with cancer progression and prognosis (He et al., 2018; Cui et al., 2020; Zhen et al., 2021). Seitz et al. (2017) originally reported that LINC00958 might interact with proteins involved in translation initiation or RNA post-transcriptional modification as a carcinogenic driving factor in bladder cancer, contributing to the aggressive cancer phenotype. Later, studies on gastric cancer (Wang et al., 2019a; Yang et al., 2021), liver cancer (Zuo et al., 2020; Lan et al., 2021), cervical cancer (Zhao et al., 2019), endometrial cancer (Chen et al., 2017; Wang et al., 2019b; Jiang et al., 2021b), oral squamous cell carcinoma (Chen et al., 2019a; Wang et al., 2020; Jiang et al., 2021a), pancreatic cancer (Chen et al., 2019c), osteosarcoma (Zhou and Mu, 2021), colorectal cancer (Sun et al., 2020; Liang et al., 2021; Luo et al., 2021), nasopharyngeal carcinoma (Chen et al., 2019b), head and neck squamous cell carcinoma (Xiong et al., 2020a; Yang et al., 2020b; Shen et al., 2021), lung cancer (Luo et al., 2019; Yang et al., 2020a; Jiang et al., 2021a; Liu et al., 2021) found that LINC00958 was aberrantly expressed. It was bound up with the clinicopathological features and prognosis of cancer. However, the small sample size and uncertain quality of LINC00958-related studies have led to controversial conclusions. Based on many studies of LINC00958 in a variety of cancers, this meta-analysis aims to provide a better clinical reference and explore the relationship between LINC00958 and clinicopathological characteristics and prognosis in cancer.

## Materials and methods

### Registration

Registration has been completed on PROSPERO (registration number: CRD42021288150).

### Search strategy

Article Collection of English articles in several databases, including PubMed, Web of Science, The Cochrane Library and Embase, until October 2021. Search terms are as follows: (“LINC00958” OR “long noncoding RNA LINC00958” OR “lncRNA LINC00958”) AND (“cancer” OR “neoplasms” OR “neoplasia” OR “tumor” OR “malignancy” OR “sarcoma” OR “melanoma” OR “carcinoma” OR “adenoma”). References to relevant studies were also hand-searched to avoid missing any valuable studies.

### Selection criteria

The inclusion criteria for this study are as follows: 1) clarify the tumor diagnosis and describe the connection between LINC00958 and survival information or clinicopathological characteristics; 2) in tumor tissues, the detection of LINC00958 expression is through real-time quantitative polymerase chain reaction (RT-qPCR); 3) hazard ratios (HRs) with 95% confidence intervals (CIs) could be obtained from the research.

The exclusion criteria for this study are as follows: 1) the article type is conference reports, reviews, editorials, letters, and case reports; 2) unable to extract original information data for the study; 3) only survival data from the database.

### Data extraction

Two reviewers independently selected eligible studies based on the above criteria and extracted data using a preset table. Disagreements between the two reviewers were resolved through consultation with a third reviewer. The general information of the study was collected, such as first author, year, country, sample size, cancer type, clinical parameters, detection methods, and overall survival (OS). If HRs and 95% CIs of OS were not provided in the article, Kaplan Meier (KM) curves were used to calculate it using the Engauge Digitizer software (Zhang et al., 2022).

### Quality assessment

Two reviewers performed independently quality evaluations based on the Newcastle-Ottawa scale (NOS). If was a difference, the third reviewer will negotiate and resolve the difference. NOS includes three aspects: group selection method, comparability, and exposure. Studies with a NOS score greater than 7 were considered high-quality studies.

### Statistical analysis

The Stata (Version 12.0) software was used to calculate the pooled odds ratios (ORs), HRs and 95% CIs. Heterogeneity was evaluated using the *I* ^2^ value. If the heterogeneity was too high (*P*_Q_ < 0.1, *I* ^2^ > 50%), a random model was used. Otherwise, the fixed model was used (*P*_Q_ > 0.1, *I* ^2^ < 50%). Next, we performed the egger’s tests and begg’s funnel plot to assess potential publication bias, and sensitivity analysis to test the robustness of the results. *p* < 0.05 was defined as statistically significant.

### Validation by using public datasets

We used Gene Expression Profiling Interactive Analysis (GEPIA), an online web-based tool based on TCGA and GTEx data, to determine the expression level of LINC00958 in various cancers (*p*-value Cutoff: 0.01). We evaluated the correlation between LINC00958 expression levels and OS in various cancer types. Furthermore, survival, somatic mutations, and RNA sequence data related to 33 cancers were downloaded from UCSC Xena (https://xena.ucsc.edu/, based on the TCGA database). Differential expression of LINC00958 in different cancers was analyzed with the “wilcox.test” method. R-package “ggpubr” was used to visualize the differential expression of LINC00958 further. Additionally, we investigated the correlation of LINC00958 expression with OS, disease-specific survival (DSS), progression-free interval (PFI), and disease-free interval (DFI) utilizing independent TCGA data. KM method and log-rank test were employed for survival analysis. Moreover, Biomarker Exploration of Solid Tumors (BEST, https://rookieutopia.com), a website that provides a comprehensive systematic analysis of the clinical relevance and biological function of genes, was employed to further analyze the prognostic value of LINC00958 in various cancers.

### Association between LINC00958 expression and immunity

Using the ESTIMATE algorithm, we calculated stromal and immune cell scores in tumor tissues. LINC00958 expression correlation with tumor microenvironment (TME) was performed using the R-package “ggpubr,” “ggplot2,” and “ggExtra” options (*p*-value Cutoff: 0.001). Additionally, we calculated tumor mutational burden (TMB) and microsatellite instability (MSI) scores based on the TCGA database of patients’ somatic mutation profiles and employed Spearman’s method to evaluate the relevance of LINC00958 expression to TMB and MSI. The results were visualized using the R-package “fmsb” option.

### Target gene prediction and signal pathway network construction

In the MEM-Multi Experiment Matrix database, we downloaded the set of genes correlated with LINC00958 expression. We then performed Gene Ontology (GO) analysis and the Kyoto Encyclopedia of Genes and Genomes (KEGG) pathway enrichment analysis using R software. Afterward, we built a visual signal pathway network through Cystoscape software.

## Results

### Literature screening

**FIGURE 1:**
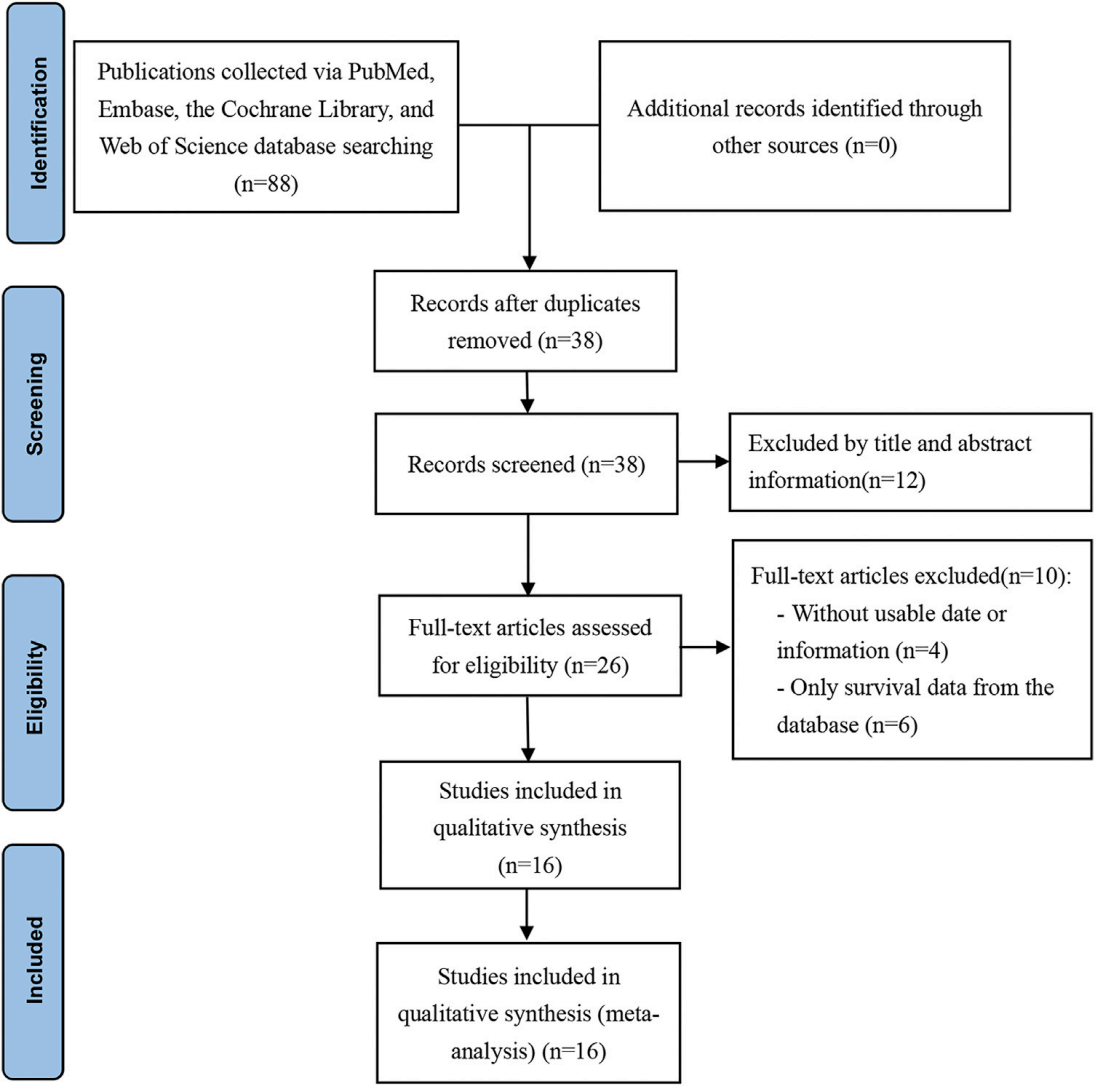
Flow diagram of this meta-analysis.

A total of 88 articles were identified using the above search strategy (PubMed = 19, The Cochrane Library = 0, Web of Science = 35, Embase = 34). 62 of these articles were removed by screening with EndNote X9 (50 duplicate articles and 12 articles were removed through title and abstract). After that, 10 articles were removed from the analysis after they were read in detail (the data cannot be extracted, or only survival data from the database). Ultimately, 16 articles were included in this study (Guo et al., 2018; He et al., 2018; Chen et al., 2019a; Wang et al., 2019a; Chen et al., 2019b; Huang et al., 2019; Zhao et al., 2019; Wang et al., 2020; Zuo et al., 2020; Jiang et al., 2021b; Jia et al., 2021; Lan et al., 2021; Liang et al., 2021; Rong et al., 2021; Yang et al., 2021; Zhou and Mu, 2021). Based on PRISMA guidelines, the retrieval process and results are shown in Figure 1.

### Quality assessment and study characteristics

A total of 16 studies were included. All of these studies were published between 2018 and 2021, with a total patient sample size of 1,121 and an average patient sample size of approximately 70.06 (range 28–200) (Table 1). The studies were as follows: one head and neck squamous cell carcinoma (Huang et al., 2019), one endometrial cancer (Jiang et al., 2021b), two gastric cancer (Wang et al., 2019a; Yang et al., 2021), one breast cancer (Rong et al., 2021), two oral squamous cell carcinoma (Chen et al., 2019a; Wang et al., 2020), one colorectal cancer (Liang et al., 2021), one glioma (Guo et al., 2018), one osteosarcoma (Zhou and Mu, 2021), one cervical cancer (Zhao et al., 2019), two liver cancer (Zuo et al., 2020; Lan et al., 2021), one nasopharyngeal carcinoma (Chen et al., 2019b), one tongue squamous cell carcinoma (Jia et al., 2021), and one bladder cancer (He et al., 2018). All studies are based on RT-qPCR to detect the expression of LINC00958 and are divided into a high and low expression group. Among the seven studies (Guo et al., 2018; He et al., 2018; Chen et al., 2019b; Huang et al., 2019; Zuo et al., 2020; Jiang et al., 2021b; Rong et al., 2021) were based on the median level of LINC00958 expression as the cutoff value, one (Wang et al., 2020) was based on the mean level of LINC00958 expression as the cutoff value, and the other eight studies were not elaboration. One study (Zuo et al., 2020) was rated 8 points, and five studies (Guo et al., 2018; He et al., 2018; Chen et al., 2019b; Jiang et al., 2021b; Rong et al., 2021) were rated 7 points. These studies were considered high-quality studies. Nine studies (Chen et al., 2019a; Wang et al., 2019a; Huang et al., 2019; Zhao et al., 2019; Wang et al., 2020; Jia et al., 2021; Liang et al., 2021; Yang et al., 2021; Zhou and Mu, 2021) were rated 6 points and one study (Lan et al., 2021) was rated 5 points. These studies were considered moderate quality studies.

**TABLE 1:** Characteristics of studies in this meta-analysis.

Study	Year	Country	Cancer type	Sample type	Total size(n)	Detection method	Cutoff	Outcome	Follow-up time (mouth)	HR statistic	NOS score
Zuo	2020	China	HCC	tissue	80	RT-qPCR	NR	OS	125	Rep	7
ChenF	2019	China	OSCC	tissue	70	RT-qPCR	NR	OS	60	SC	7
ChenM	2019	China	NPC	tissue	59	RT-qPCR	NR	OS	50	SC	7
Guo	2018	China	Glioma	tissue	35	RT-qPCR	NR	OS	50	SC	7
He	2018	China	Bladder cancer	tissue	140	RT-qPCR	median	OS	100	SC	8
Huang	2019	China	HNSCC	tissue	48	RT-qPCR	NR	NR	NR	NR	6
Jiang	2021	China	EC	tissue	40	RT-qPCR	median	OS	100	SC	8
Liang	2021	China	CRC	tissue	63	RT-qPCR	NR	OS	90	SC	7
Rong	2021	China	BC	tissue	30	RT-qPCR	NR	OS	60	SC	7
WangW	2019	China	GC	tissue	200	RT-qPCR	NR	OS	50	SC	7
YangD	2021	China	GC	tissue	46	RT-qPCR	NR	OS	60	SC	7
Zhao	2019	China	CC	tissue	57	RT-qPCR	NR	OS	60	SC	7
Zhou	2021	China	OSA	tissue	63	RT-qPCR	NR	OS	60	SC	7
Lan	2021	China	HCC	tissue	49	RT-qPCR	NR	NR	NR	NR	6
Jia	2021	China	TSCC	tissue	113	RT-qPCR	NR	NR	NR	NR	6
WangZ	2020	China	OSCC	tissue	28	RT-qPCR	NR	NR	NR	NR	6

HR, hazard ratio; HCC, hepatocellular carcinoma; OSCC, oral squamous cell carcinoma; NPC, nasopharyngeal carcinoma; HNSCC, head and neck squamous cell carcinoma; EC, endometrial cancer; CRC, colorectal cancer; BC, breast cancer; GC, gastric cancer; CC, cervical cancer; OSA, osteosarcoma; TSCC, tongue squamous cell carcinoma; NR, no report; OS, overall survival; Rep, report; SC, survival curve; RT-qPCR, real-time quantitative polymerase chain reaction.

### Association between LINC00958 and overall survival

**FIGURE 2:**
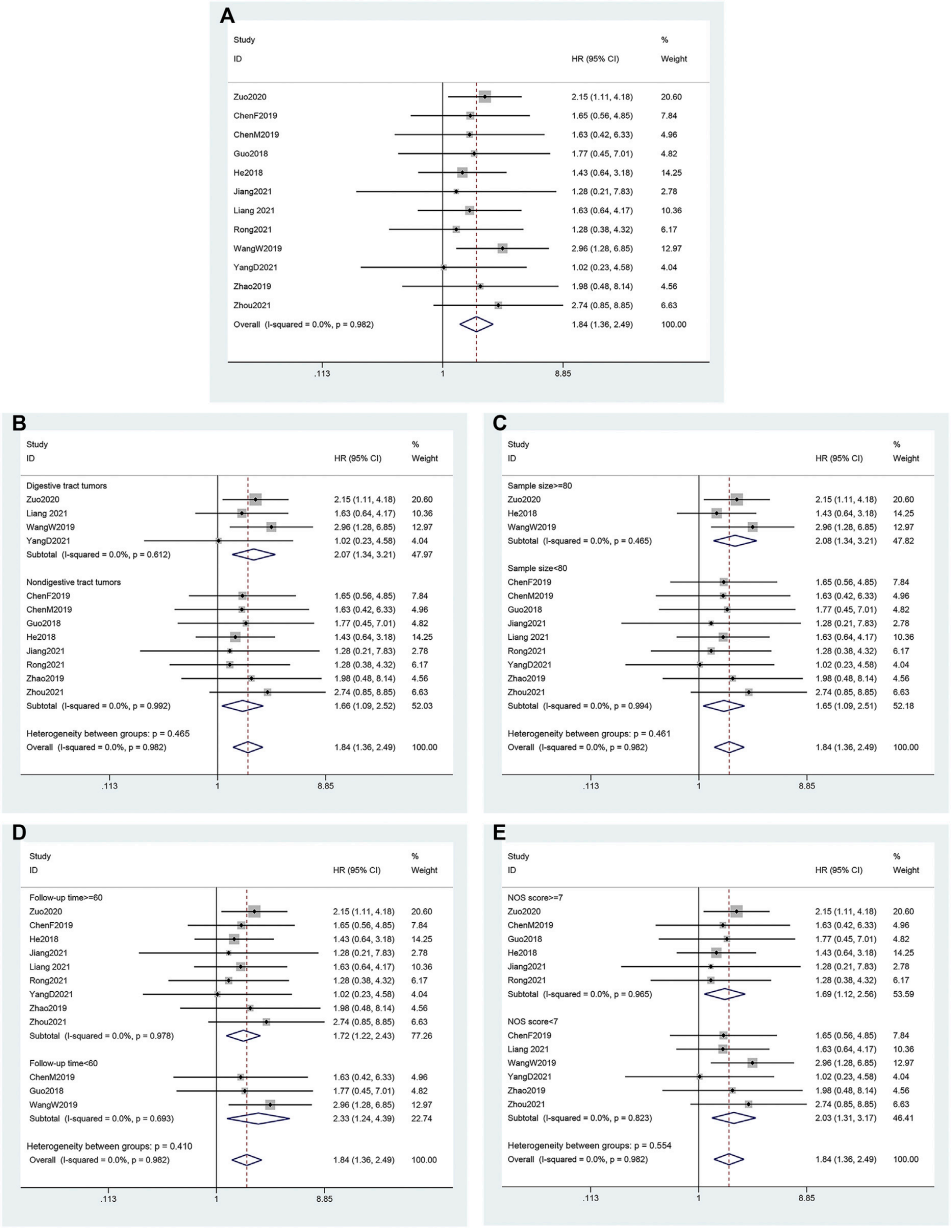
Association of LINC00958 expression with overall survival. **(A)** Forest plots for an association of LINC00958 expression with overall survival. **(B)** Subgroup analysis stratified by cancer type. **(C)** Subgroup analysis stratified by sample size. **(D)** Subgroup analysis stratified by follow-up time. **(E)** Subgroup analysis stratified by NOS score.

Twelve studies (Guo et al., 2018; He et al., 2018; Chen et al., 2019a; Wang et al., 2019a; Chen et al., 2019b; Zhao et al., 2019; Zuo et al., 2020; Jiang et al., 2021b; Liang et al., 2021; Rong et al., 2021; Yang et al., 2021; Zhou and Mu, 2021) reported the association between differential LINC00958 expression and OS, so we extracted data (pooled HRs and 95% CIs) from 883 patients from 12 studies. The fixed-effects model (*I* ^2^ = 0.0%; *P*_Q_ = 0.982) analysis showed that the high expression of LINC00958 was significantly correlated with OS (HR = 1.84; 95% CI: 1.36–2.49; *p* < 0.001; Figure 2A). Furthermore, we performed a subgroup analysis using the fixed-effects model based on the cancer type (digestive tract tumors and nondigestive tract tumors), sample size (*n* ≥ 80 or *n* < 80), follow-up time (≥60 months or <60 months), and NOS score (NOS scores ≥7 or <7) (Table 2). There is a significant correlation between each group and the expression of LINC00958, which is consistent with the results shown above. The data are as follows: digestive tract tumors (HR = 2.07; 95% CI: 1.34–3.21; *p* = 0.001) and non-digestive tract tumors (HR = 1.66; 95% CI: 1.09–2.52; *p* = 0.018) (Figure 2B); sample sizes greater than or equal to 80 (HR = 2.08; 95% CI: 1.34–3.21; *p* = 0.001) and less than 80 (HR = 1.65; 95% CI: 1.09–2.51; *p* = 0.018) (Figure 2C); follow-up was at least 60 months (HR = 1.72; 95% CI: 1.22–2.43; *p* = 0.002) and less than 60 months (HR = 2.33; 95% CI: 1.24–4.39; *p* = 0.009) (Figure 2D); NOS score greater than or equal to 7 (HR = 1.69; 95% CI: 1.12–2.56; *p* = 0.012) and less than 7 (HR = 2.03; 95% CI: 1.31–3.17; *p* = 0.002) (Figure 2E).

**TABLE 2:** Subgroup meta-analysis of pooled HRs for OS.

Stratified analysis	Studies (n)	Number of patients	Pooled HR (95% CI)	*p*-value	Heterogeneity	
					*I* ^2^ (%)	*p*-value
Cancer type						
Digestive tract tumors	4	389	2.07 (1.34–3.21)	0.001	0	0.612
Nondigestive tract tumors	8	494	1.66 (1.09–2.52)	0.018	0	0.992
Sample size						
≥80	3	420	2.08 (1.34–3.21)	0.001	0	0.465
<80	9	463	1.65 (1.09–2.51)	0.018	0	0.994
Follow-up time						
≥60	9	589	1.72 (1.22–2.43)	0.002	0	0.978
<60	3	294	2.33 (1.24–4.39)	0.009	0	0.693
NOS score						
≥7	6	384	1.69 (1.12–2.56)	0.012	0	0.965
<7	6	499	2.03 (1.31–3.17)	0.002	0	0.823

### Association between LINC00958 and clinicopathological features

**FIGURE 3:**
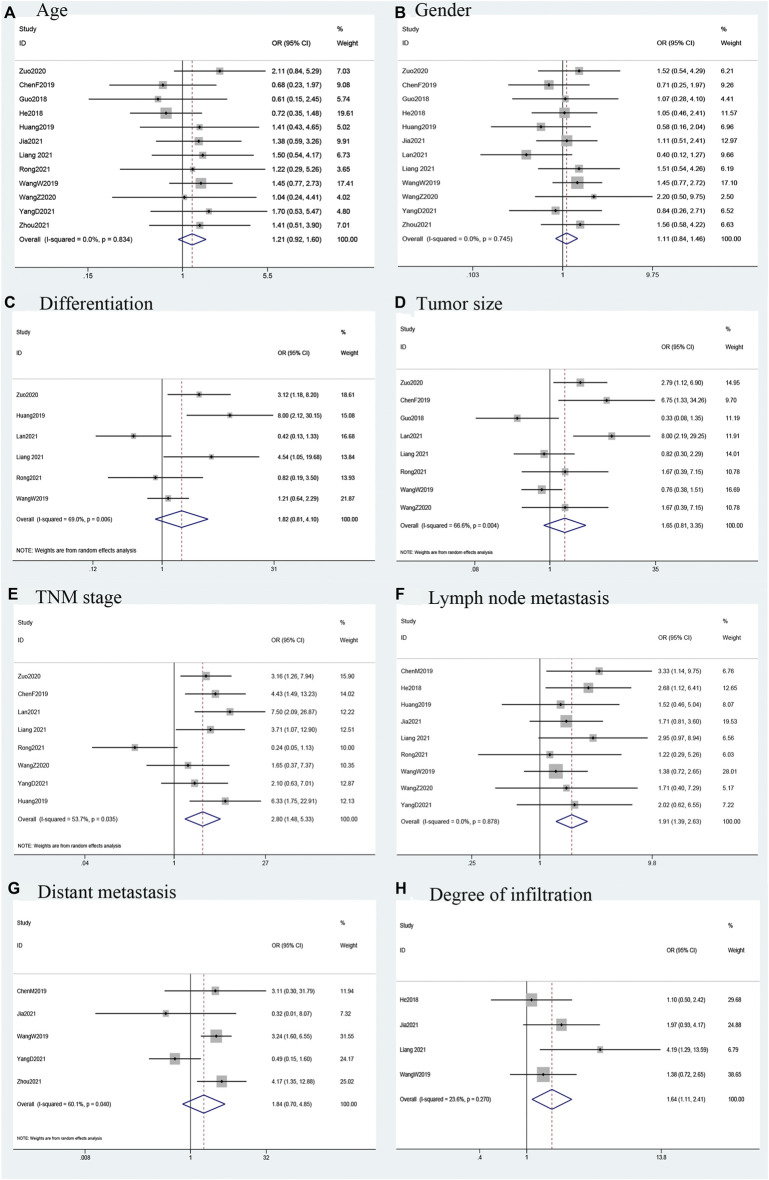
(Continued).

Fourteen studies that explored the relationship between the expression of LINC00958 in malignant tumors and clinical case characteristics. This relationship is shown in Figure 3 and Table 3. A total of 8 studies (Chen et al., 2019a; Huang et al., 2019; Wang et al., 2020; Zuo et al., 2020; Lan et al., 2021; Liang et al., 2021; Rong et al., 2021; Yang et al., 2021) (415 patients) reported the relationship between the expression of LINC00958 and tumor-node-metastases (TNM). We used a random-effects model (*I* ^2^ = 53.7%; *P*_Q_ = 0.035) analysis to show that the high expression of LINC00958 was significantly correlated with advanced TNM stage (OR = 2.80; 95% CI: 1.48–5.33; *p* = 0.002). We extracted the data of 729 patients from 9 studies (He et al., 2018; Wang et al., 2019a; Chen et al., 2019b; Huang et al., 2019; Wang et al., 2020; Jia et al., 2021; Liang et al., 2021; Rong et al., 2021; Yang et al., 2021), and used the fixed-effect model (*I* ^2^ = 0.0%; *P*_Q_ = 0.878) to show that the high expression of LINC00958 was significantly correlated with positive lymph node metastasis (LNM) (OR = 1.91; 95% CI: 1.39–2.63; *p* < 0.001). Data from 516 patients was extracted from 4 studies (He et al., 2018; Wang et al., 2019a; Jia et al., 2021; Liang et al., 2021) and we used the fixed-effect model (*I* ^2^ = 23.6%; *P*_Q_ = 0.270) to showed that the differential expression of LINC00958 was associated with degree of infiltration (OR = 1.64; 95% CI: 1.11–2.41; *p* = 0.013). However, we separately extracted data and analyzed that there was no significant correlation between age (OR = 1.21; 95% CI: 0.92–1.60; *p* = 0.174), sex (OR = 1.11; 95% CI: 0.84–1.46; *p* = 0.483), tumor size (OR = 1.65; 95% CI: 0.81–3.35; *p* = 0.166), distant metastasis (OR = 1.84; 95% CI: 0.70–4.85; *p* = 0.220), and tumor differentiation degree (OR = 1.82; 95% CI: 0.81–4.10; *p* = 0.146) with LINC00958 expression.

**TABLE 3:** Association of LINC00958 expression with clinicopathological features.

Clinicopathological parameters	Patients (n)	OR (95%CI)	*p*-value	Heterogeneity (*I* ^*2*^, *P*)	Model
Age (elderly vs. nonelderly)	917	1.21 (0.92–1.60)	0.174	0.0%, 0.834	Fixed
Sex (male vs. female)	926	1.11 (0.84–1.46)	0.483	0.0%, 0.745	Fixed
Lymph node metastasis (positive vs. negative)	729	1.91 (1.39–2.63)	<0.001	0.0%, 0.878	Fixed
Tumor size (big vs. small)	546	1.65 (0.81–3.35)	0.166	66.6%, 0.004	Random
TNM (III + IV vs. I + II)	415	2.80 (1.48–5.33)	0.002	53.7%, 0.035	Random
Distant metastasis (presence vs. absence)	481	1.84 (0.70–4.85)	0.220	60.1%, 0.040	Random
Differentiation (pool + moderate vs. well)	470	1.82 (0.81–4.10)	0.146	69.0%,0.006	Random
Degree of infiltration (III + IV vs. I + II)	516	1.64 (1.11–2.41)	0.013	23.6%,0.270	Fixed

### Risk of bias and sensitivity analysis

**FIGURE 4:**
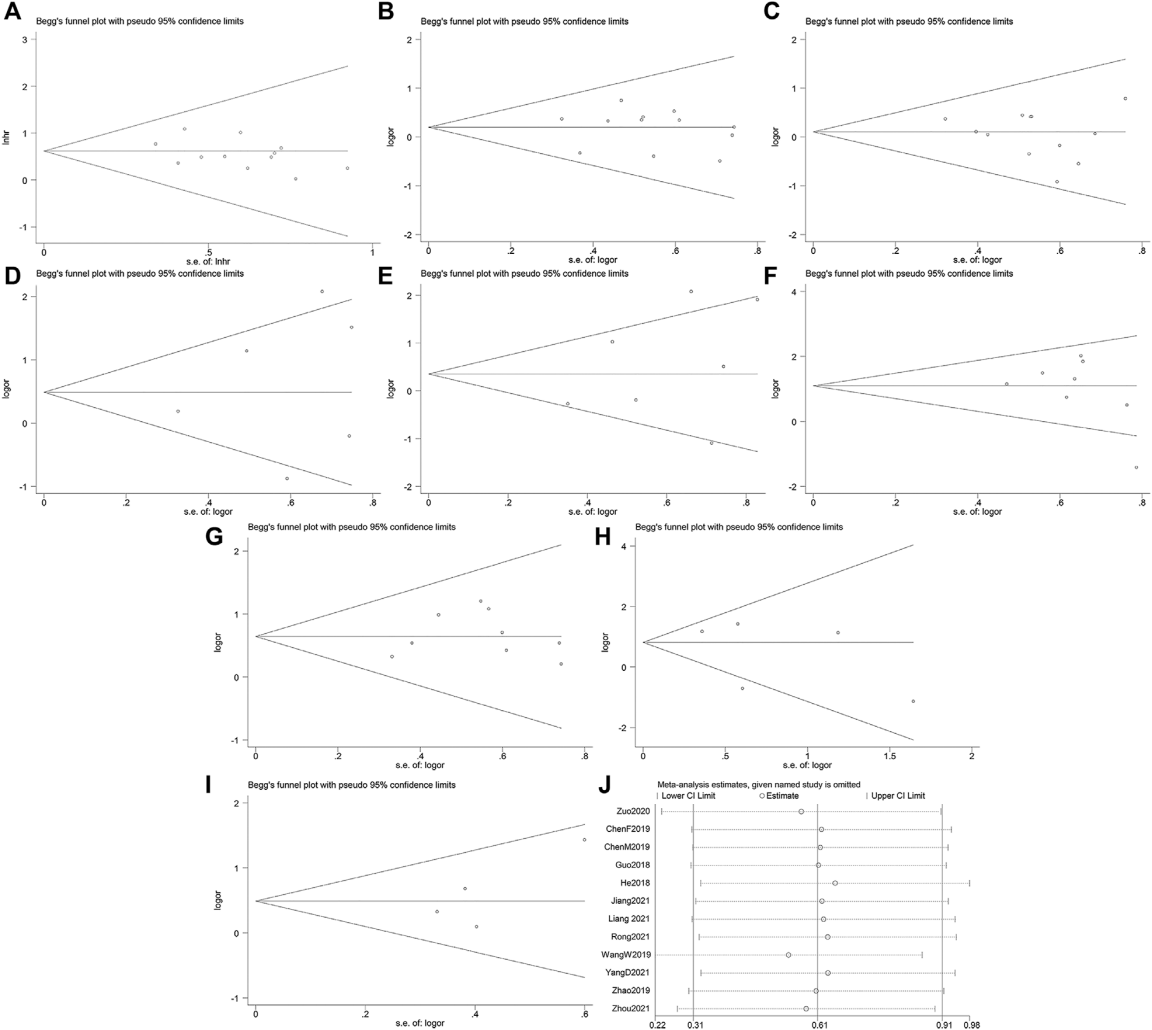
Begg’s funnel plots and sensitivity analysis. **(A)** Begg’s funnel plot for OS. **(B)** Begg’s funnel plot for age. **(C)** Begg’s funnel plot for sex. **(D)** Begg’s funnel plot for differentiation. **(E)** Begg’s funnel plot for tumor size. **(F)** Begg’s funnel plot for the TNM stage. **(G)** Begg’s funnel plot for lymph node metastasis. **(H)** Begg’s funnel plot for distant metastasis. **(I)** Begg’s funnel plot for degree of infiltration. **(J)** Sensitivity analysis for studies about OS.

The egger’s test and begg’s funnel plot found no significant evidence of publication bias. Respectively as follows: OS (*P* > |t| = 0.181; Figure 4A), age (*P* > |t| = 0.798; Figure 4B), sex (*P* > |t| = 0.347; Figure 4C), tumor differentiation degree (*P* > |t| = 0.584; Figure 4D), tumor size (*P* > |t| = 0.327; Figure 4E), TNM (*P* > |t| = 0.250; Figure 4F), LNM (*P* > |t| = 0.537; Figure 4G), distant metastasis (*P* > |t| = 0.473; Figure 4H), degree of infiltration (*P* > |t| = 0.237; Figure 4I). In order to evaluate the impact of individual studies on the overall study, we performed sensitivity analysis by reducing the number of qualified studies one at a time (Figure 4J). Our analysis did not show any evidence of any individual study substantially biasing the results of our analysis.

### Validation of LINC00958 expression in public databases

**FIGURE 5:**
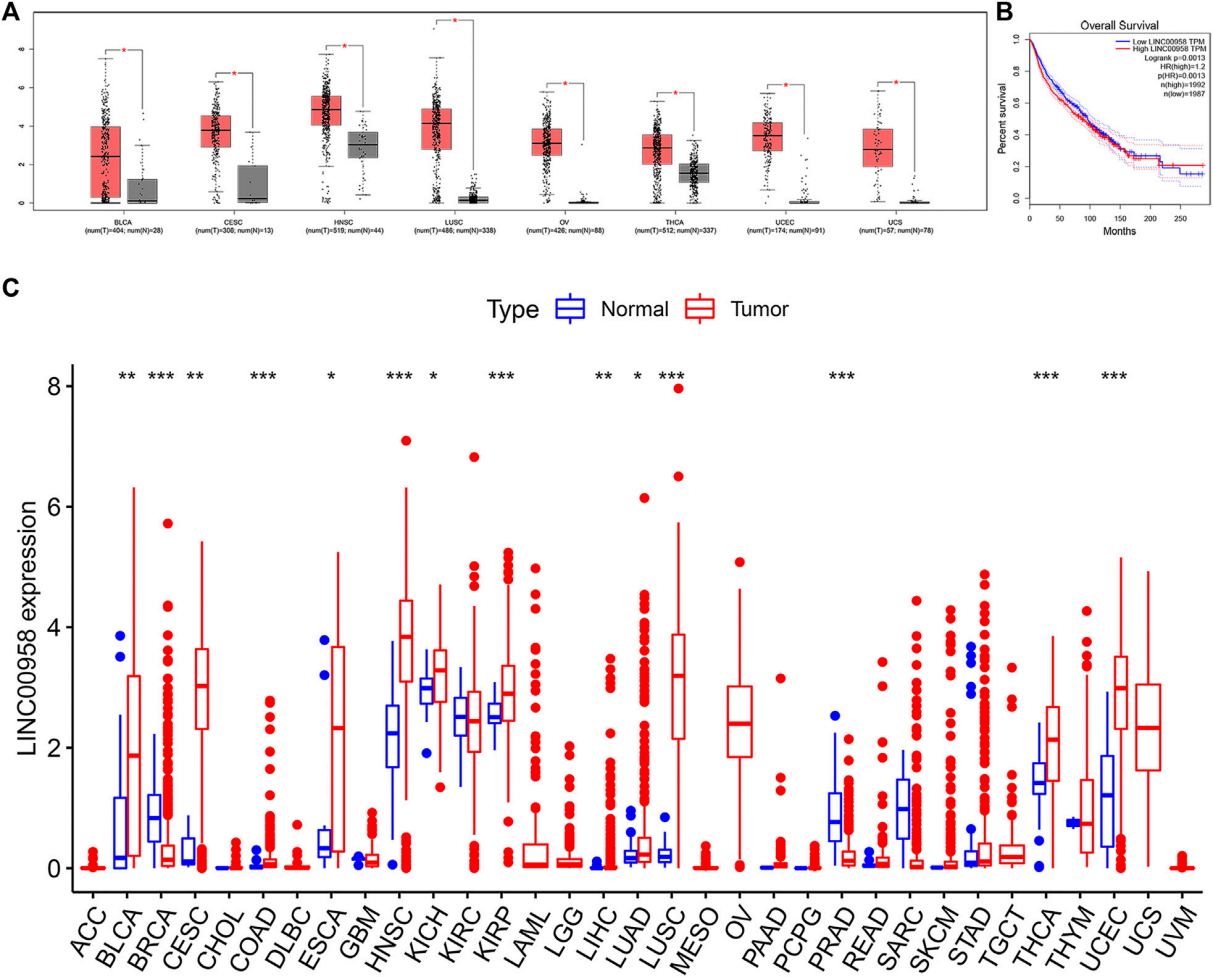
Validation of the role of LINC00958 in human cancers in public databases. **(A)** The expression of LINC00958 in human cancers (red box) and normal tissues (gray box) based on the TCGA and GTEx databases (|Log2FC| > 1 and *p* < 0.01); **(B)** Overall survival (OS) plots based on LINC00958 expression in nine types of cancer (*n* = 3,979); **(C)** LINC00958 expression levels in pan-cancer from TCGA data. *p < 0.05, **p < 0.01, and ***p < 0.001.

We further validated our results using GEPIA data. We found that high expression of LINC00958 can be observed in a variety of cancers, such as uterine corpus endometrial carcinoma (UCEC), cervical squamous cell carcinoma and endocervical adenocarcinoma (CESC), bladder urothelial carcinoma (BLCA), HNSC, lung squamous cell carcinoma (LUSC), ovarian serous cystadenocarcinoma (OV), thyroid carcinoma (THCA), and uterine carcinosarcoma (UCS) (Figure 5A). We aggregated survival data for nine cancers, including BLCA, CESC, HNSC, breast invasive carcinoma (BRCA), colon adenocarcinoma (COAD), glioma (LGG), liver hepatocellular carcinoma (LIHC), stomach adenocarcinoma (STAD), and UCEC. Ultimately, 3,979 patients were divided into two groups (high and low expression group) based on the median LINC00958 expression. Not surprisingly, our results were consistent with our prior analysis. The OS of the high expression group was shorter, which further verified that the high expression of LINC00958 was associated with a poor prognosis (Figure 5B).

**FIGURE 6:**
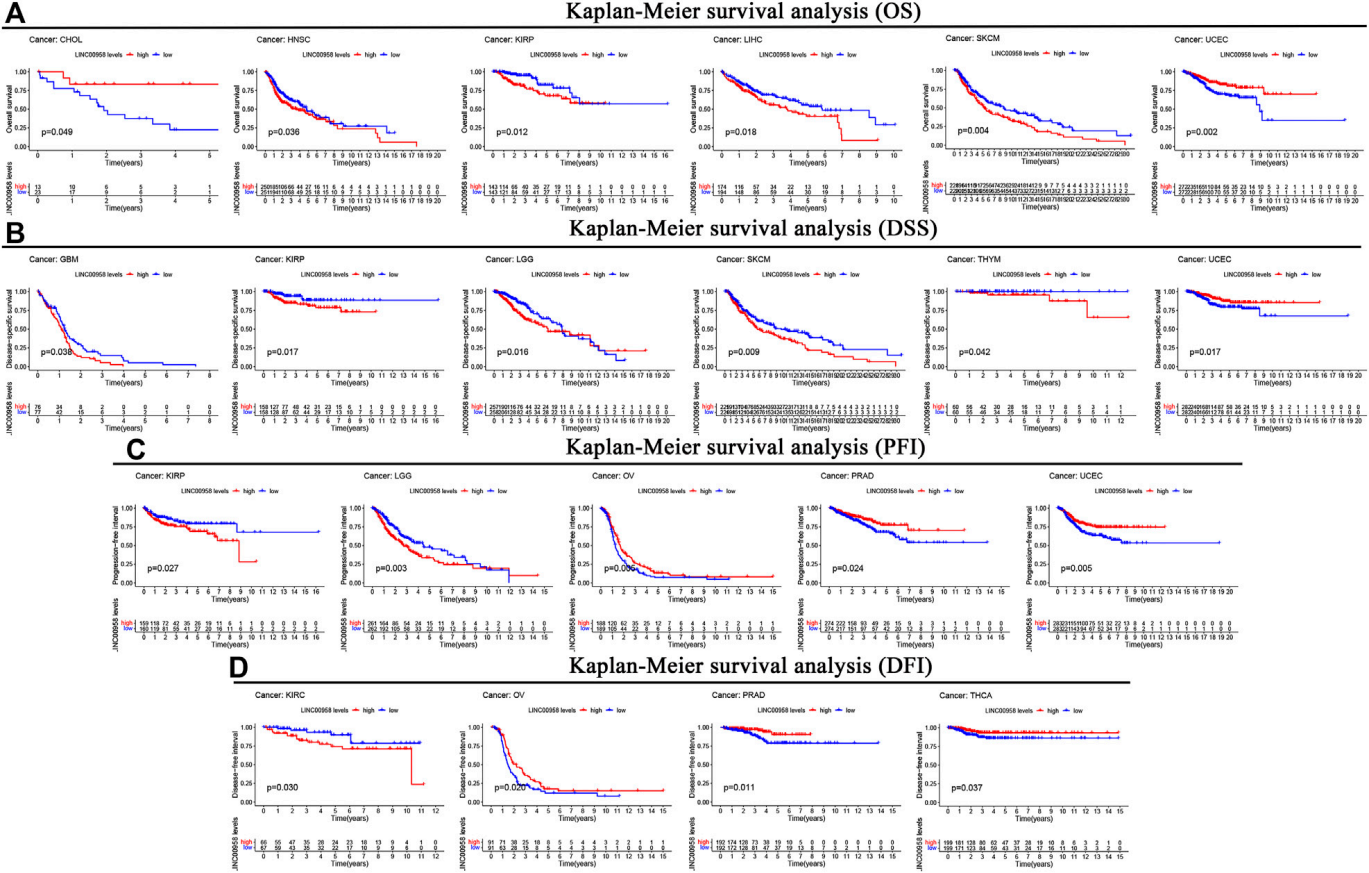
Kaplan-Meier survival curves comparison of high and low expression of LINC00958 for different cancer types. **(A)** OS. **(B)** DSS. **(C)** PFI. **(D)** DFI.

We further explored the expression of LINC00958 in various cancers and the association of LINC00958 expression with prognosis utilizing an independent TCGA dataset. Our findings demonstrated that LINC00958 is overexpressed in certain specific cancer types, including BLCA, CESC, COAD, esophageal carcinoma (ESCA), HNSC, kidney chromophobe (KICH), kidney renal papillary cell carcinoma (KIRP), LIHC, lung adenocarcinoma (LUAD), LUSC, THCA, and UCEC (Figure 5C). In contrast, LINC00958 expression was low in BRCA and prostate adenocarcinoma (PRAD) (Figure 5C). KM curves show that LINC00958 expression was associated with prognosis in some cancer patients (Figure 6). Of these, LINC00958 was associated with shorter OS in eight cancers, including HNSC (OS: *p* = 0.036), KIRP (OS: *p* = 0.012; DSS: *p* = 0.017; PFI: *p* = 0.027), LIHC (OS: *p* = 0.018), skin cutaneous melanoma (SKCM) (OS: *p* = 0.004; DSS: *p* = 0.009), glioblastoma multiforme (GBM) (DSS: *p* = 0.038), LGG (DSS: *p* = 0.016; PFI: *p* = 0.003), thymoma (THYM) (DSS: *p* = 0.042), and kidney renal clear cell carcinoma (KIRC) (DFI: *p* = 0.030). In contrast, LINC00958 was associated with longer OS in five other cancers, including cholangiocarcinoma (CHOL) (OS: *p* = 0.049), UCEC (OS: *p* = 0.002; DSS: *p* = 0.017; PFI: *p* = 0.005), OV (PFI: *p* = 0.005; DFI: *p* = 0.020), PRAD (PFI: *p* = 0.024; DFI: *p* = 0.011), and THCA (DFI: *p* = 0.037).

**FIGURE 7:**
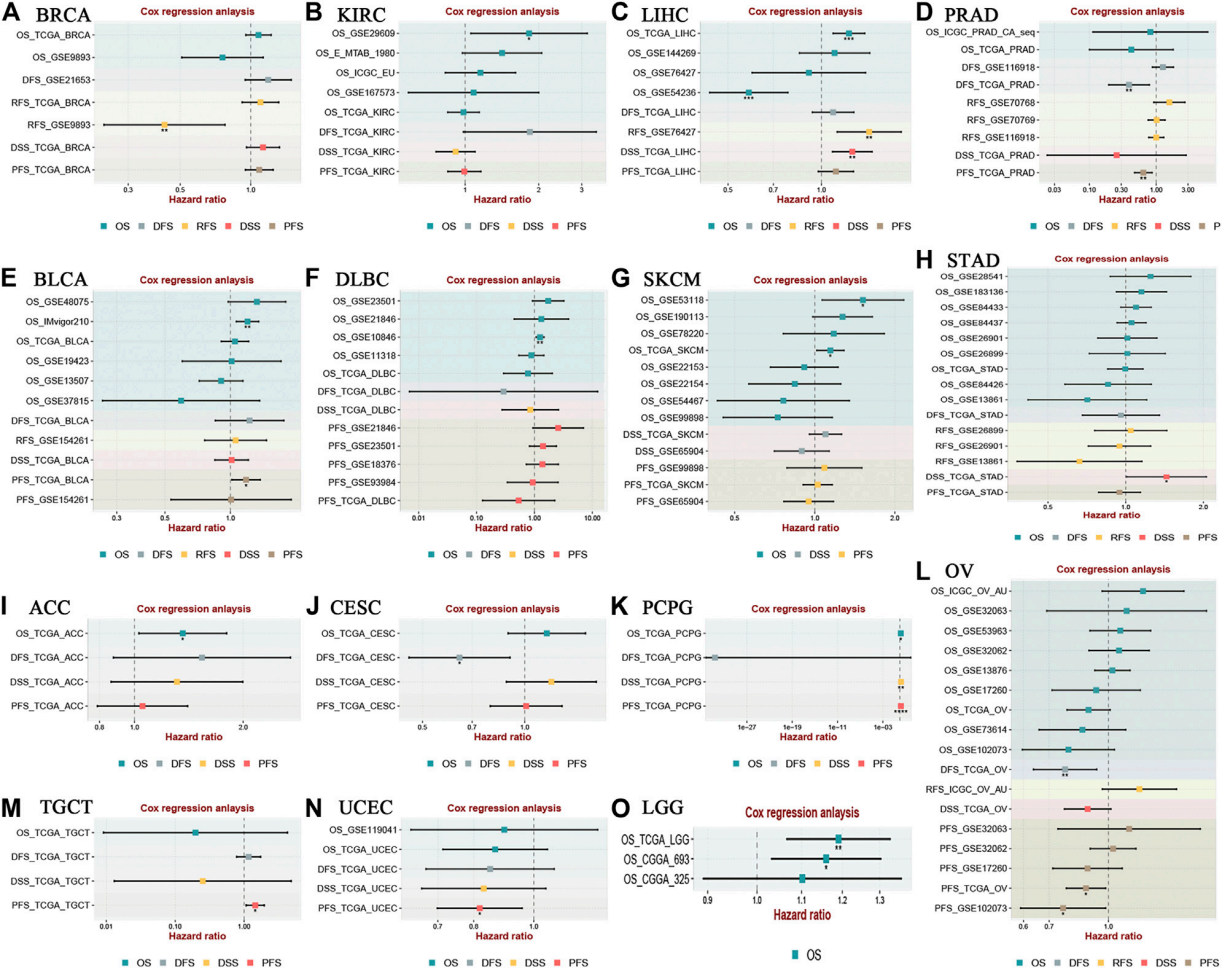
Relevance analysis of LINC00958 expression with survival using the Cox method for different types of cancers in BEST (https://rookieutopia.com). Cox regression analysis of **(A)** BRCA, **(B)** KIRC, **(C)** LIHC, **(D)** PRAD, **(E)** BLCA, **(F)** DLBC, **(G)** SKCM, **(H)** STAD, **(I)** ACC, **(J)** CESC, **(K)** PCPG, **(L)** OV, **(M)** TGCT, **(N)** UCEC, **(O)** LGG. *p < 0.05, **p < 0.01, and ***p < 0.001.

In addition, the prognostic value of LINC00958 in various cancers was further explored in the BEST online database using Cox regression model (Figure 7). The findings indicated that LINC00958 overexpression was significantly related to poor prognosis in KIRC, LIHC, BLCA, lymphoid neoplasm diffuse large B-cell lymphoma (DLBC), SKCM, STAD, adrenocortical carcinoma (ACC), testicular germ cell tumors (PCPG), testicular germ cell tumors (TGCT), and LGG. In contrast, LINC00958 overexpression was strongly related to favorable prognosis in BRCA, PRAD, CESC, OV, and UCEC.

### Correlation analysis on tumor mutational burden and microsatellite instability

**FIGURE 8:**
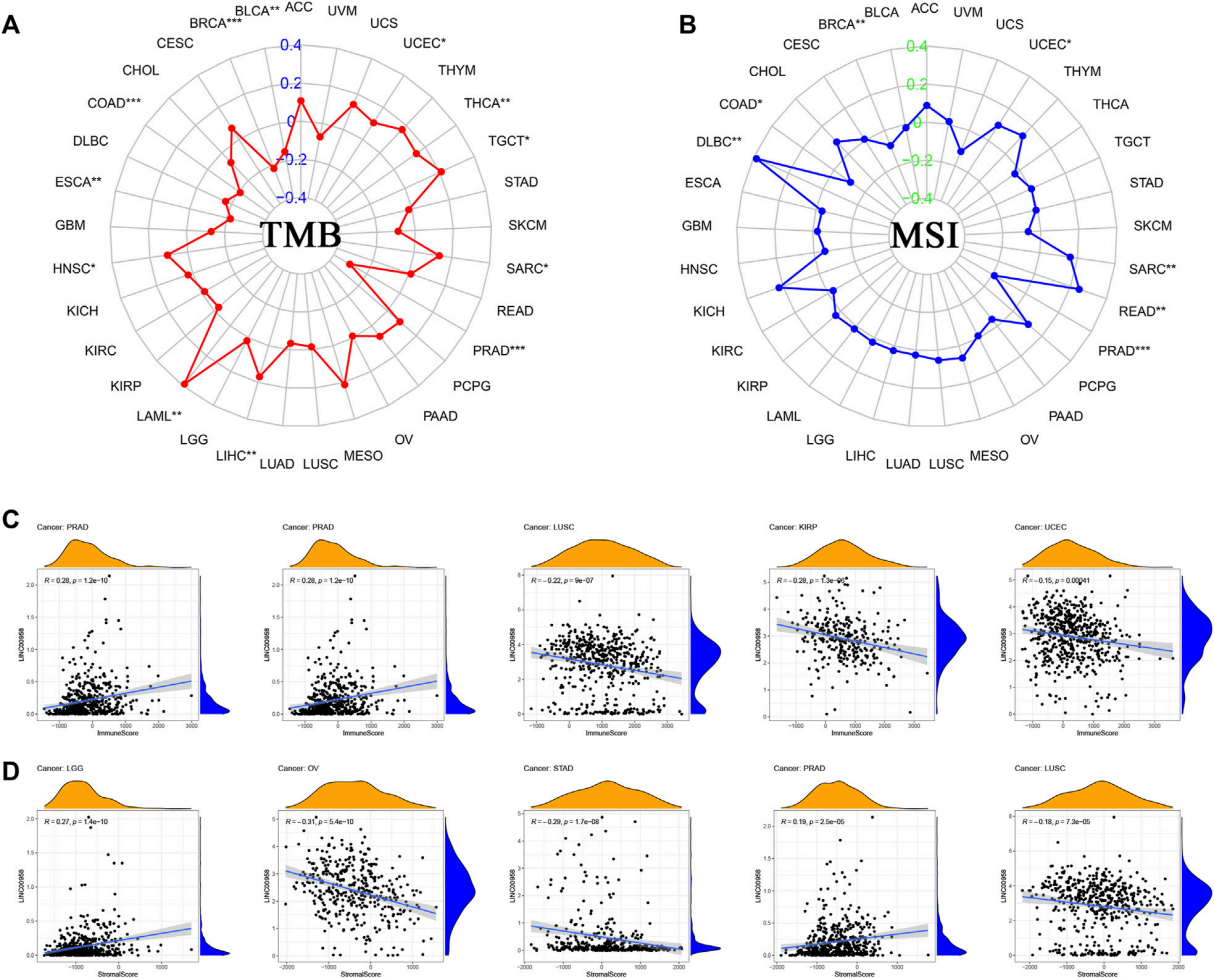
Correlations between LINC00958 expression and immunity, including TMB, MSI, stromal score, and immune score in cancers. **(A)** The radar chart showed the correlation between TMB and LINC00958 expression in pan-cancer. The red curve indicates the correlation coefficient, and the blue value indicates the range. **(B)** The radar chart showed the correlation between MSI and LINC00958 expression in pan-cancer. The blue curve indicates the correlation coefficient, and the green value indicates the range. *p < 0.05, **p < 0.01, and ***p < 0.001. **(C)** Correlation of LINC00958 expression with the immune score in pan-cancer. **(D)** Correlation of LINC00958 expression with the stromal score in pan-cancer.

We investigated the correlation of TMB/MSI with LINC00958 expression. The findings demonstrated that there was a remarkable positive correlation of LINC00958 expression with TMB in HNSC (*p* = 0.017), acute myeloid leukemia (LAML, *p* = 0.002), LIHC (*p* = 0.001), sarcoma (SARC, *p* = 0.041), TGCT (*p* = 0.012), THCA (*p* = 0.002), UCEC (*p* = 0.016), while a significant negative correlation in BLCA (*p* = 0.002), BRCA (*p* < 0.001), COAD (*p* < 0.001), ESCA (*p* = 0.005), PRAD (*p* < 0.001) (Figure 8A). Moreover, we found that LINC00958 expression was positively correlated with MSI in DLBC (*p* = 0.007), rectum adenocarcinoma (READ, *p* = 0.002), SARC (*p* = 0.010), UCEC (*p* = 0.028), and negatively correlated with MSI in BRCA (*p* = 0.006), COAD (*p* = 0.026), PRAD (*p* < 0.001) (Figure 8B).

### Correlation of LINC00958 expression with tumor microenvironment

Stromal and immune cell scores of 33 tumors were calculated with ESTIMATE. We determined the correlation of LINC00958 expression levels with these two scores. Our findings showed that the top five cancers with the strongest correlation of LINC00958 with immune score were PRAD, STAD, LUSC, KIRP, and UCEC (Figure 8C). The top five cancers with the strongest correlation of LINC00958 with stromal score were LGG, OV, STAD, PRAD, and LUSC (Figure 8D).

### Analysis of LINC00958-related genes

**FIGURE 9:**
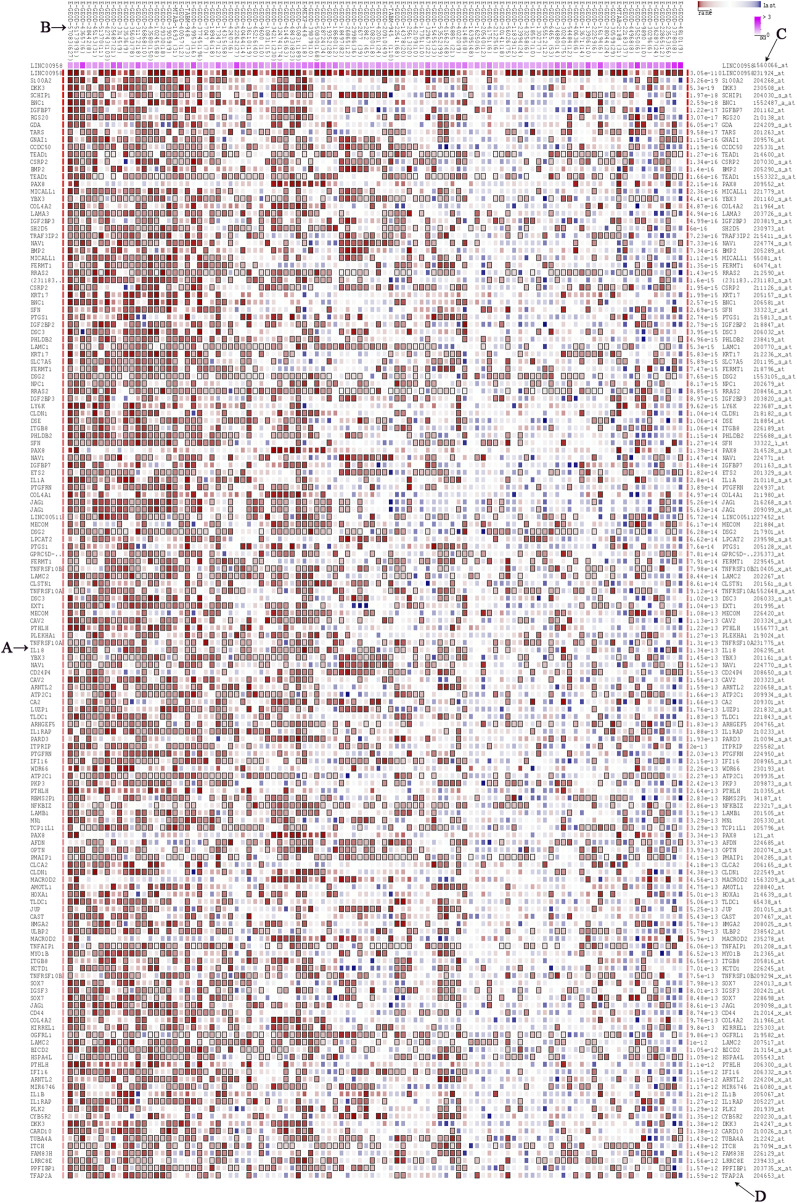
The top 150 predicted target genes of LINC00958 by using Multi Experiment Matrix (MEM, http://biit.cs.ut.ee/mem/) website. **(A)** predicted target genes; **(B)** Single experimental data set; **(C)** Gene probes; **(D)** *p* values.

**FIGURE 10:**
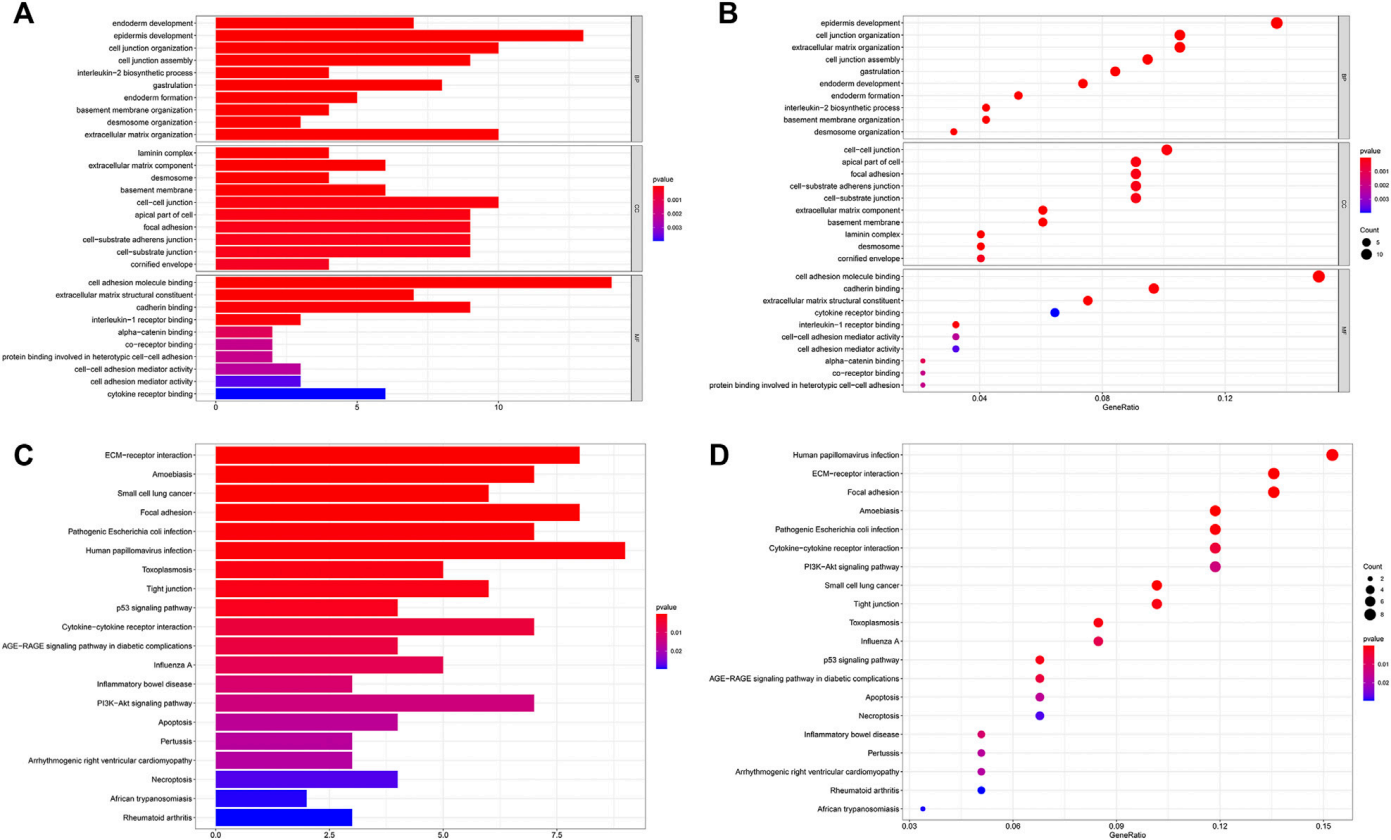
GO terms and the KEGG pathway. **(A)** Histogram presentation of the top 10 positions of GO in terms of target genes in biological processes (BP), cellular components (CC) and molecular functions (MF) of biology; **(B)** Bubble chart of the top 10 positions of GO in terms of target genes in BP, CC, MF; **(C)** Histogram presentation of pathways related to the differentially expressed genes by the KEGG analysis; **(D)** Bubble chart of pathways related to the differentially expressed genes by the KEGG analysis.

**FIGURE 11:**
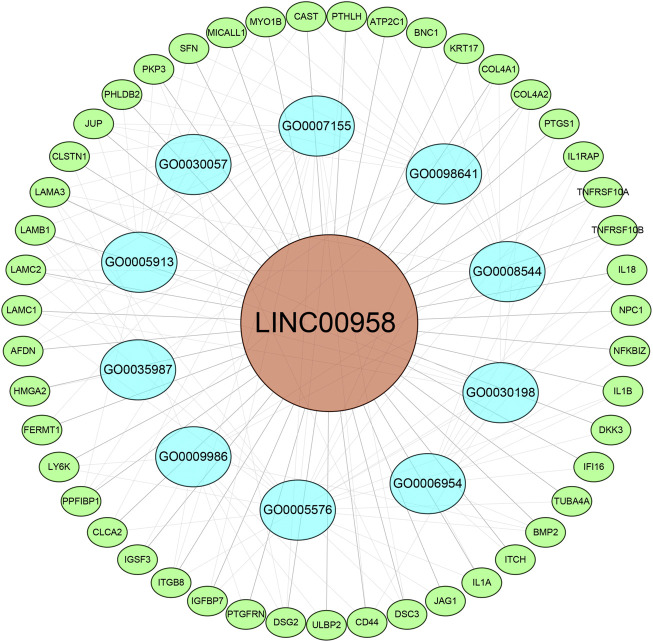
Differentially expressed gene interaction network analysis. Green nodes represent target genes, and blue nodes represent the related pathway. As indicated in red, LINC00958 is localized at the center of the network.

In the MEM-Multi Experiment Matrix database, we screened the first 150 genes co-expressed with LINC00958 and then analyzed their correlation (Figure 9). We found S100A2, DKK3 and SCHIP1 to be associated with elevated expression of LINC00958. It is one of the first three different predicted target genes. After that, we analyzed the GO and KEGG pathways (Figure 10) (Table4). GO analysis results indicated that LINC00958 may be involved in the organization of the extracellular matrix organization, cell adhesion molecule binding, and interleukin−2 biosynthetic process. The results of the KEGG pathway analysis revealed that LINC00958 may be chiefly engaged in the P53 signaling pathway, human papillomavirus infection, and ECM−receptor interaction. In addition, we used Cystoscape to establish a signal pathway network to understand the molecular mechanism (Figure 11).

**TABLE 4:** Gene ontology analysis of LINC00958-related genes.

GO number	Description	Genes	*p*-value
GO:0007155	cell adhesion	JUP, CISTN1, LAMA3, LAMB1, LAMC2, LAMC1, AFDN, FERMT1, PPFIBP1, CLCA2, ITGB8, IGFBP7, DSG2, CD44, and DSC3	1.92E-07
GO:0006954	inflammatory response	IL1A, ITCH, BMP2, IFI16, IL1B, NFKBIZ, IL18, TNFRSF10B, TNFRSF10A, IL1RAP, and PTGS1	4.24E-05
GO:0030198	extracellular matrix organization	COL4A2, COL4A1, LAMA3, ITGB8, LAMB1, LAMC2, LAMC1, and CD44	0.000101
GO:0008544	epidermis development	KRT17, BNC1, LAMA3, LAMC2, ATP2C1, and PTHLH	0.000103
GO:0098641	cadherin binding involved in cell-cell adhesion	CAST, AFDN, PPFIBP1, MYO1B, JUP, MICALL1, SFN, PKP3, and PHLDB2	0.000135
GO:0030057	desmosome	JUP, DSG2, PKP3, and DSC3	0.000265
GO:0005913	cell-cell adherents’ junction	CAST, AFDN, PPFIBP1, MYO1B, JUP, MICALL1, SFN, PKP3, and PHLDB2	0.000306
GO:0035987	endodermal cell differentiation	COL4A2, LAMA3, HMGA2, and LAMB1	0.000423
GO:0009986	cell surface	LY6K, BMP2, IGSF3, CISTN1, ITGB8, TNFRSF10B, DSG2, TNFRSF10A, PTGFRN, ULBP2, and CD44	0.000568
GO:0005576	extracellular region	LY6K, JAG1, LAMA3, IL18, LAMB1, LAMC2, IL1RAP, LAMC1, TUBA4A, PTHLH, DKK3, IL1A, BMP2, NPC1, COL4A2, COL4A1, IL1B, CLCA2, IGFBP7, and DSC3	0.00065

## Discussion

Research on lncRNAs is becoming increasingly popular due to the potential to diagnose cancer earlier and improve cancer treatment. LINC00958 has recently received increased attention from the research community. We conducted this study to explore further the relationship between the expression of LINC00958 and the clinicopathological features and prognosis of cancer. Our results showed that the overexpression of LINC00958 was significantly correlated with the adverse outcomes of cancer patients. Among the various clinicopathological characteristics, our study found that high expression of LINC00958 in cancer patients was significantly correlated with positive LNM, advanced TNM stage, and advanced degree of infiltration. However, no significant correlation was observed with the high expression of LINC00958 in age, sex, tumor metastasis, tumor size, and degree of tumor differentiation. It could be clearly observed that the overexpression of LINC00958 was significantly related to lower OS. In addition, we conducted a subgroup analysis of OS. Whether grouped by tumor type, sample size, group by follow-up time, or NOS score, each group had a significant correlation. Not only that, but no obvious heterogeneity was observed among the studies, which further verified the significant correlation between the high expression of LINC00958 and poor OS in cancer patients.

Furthermore, we explored the expression of LINC00958 in public pan-cancer datasets and its relationship to test for an association with prognosis. We found that LINC00958 was overexpressed in certain cancers, including BLCA, CESC, COAD, ESCA, HNSC, KICH, KIRP, LIHC, LUAD, LUSC, THCA, OV, UCEC, and UCS, while it was under-expressed in BRCA and PRAD. However, one study reported that LINC00958 was significantly overexpressed in BRCA (Rong et al., 2021), while the expression of LINC00958 in PRAD has not been reported. The opposite results may be related to the low number of normal tissues in the TCGA database, so the expression levels of LINC00958 in BRCA and PRAD still need further verification. Moreover, by KM method, we also found that LINC00958 affected the prognosis of cancer patients as a deleterious factor in eight cancers, including HNSC, KIRP, LIHC, SKCM, GBM, LGG, THYM and KIRC, and as a protective factor in CHOL, UCEC, OV, PRAD and THCA species of cancer. By Cox regression analysis, we found that LINC00958 was a detrimental prognostic factor in KIRC, LIHC, BLCA, DLBC, SKCM, STAD, ACC, PCPG, TGCT, and LGG. While LINC00958 was a favorable prognostic factor in BRCA, PRAD, CESC, OV, and UCEC. Integrating the results of KM analysis and Cox regression analysis, LINC00958 may have better predictive value in LIHC, SKCM, LGG, KIRC, OV, PRAD, UCEC. Altogether, these findings suggested that LINC00958 may be potentially used as a new biomarker to predict the prognosis of cancer patients.

Several studies have reported that LINC00958 is correlated with the glycolytic metabolism of tumors, and plays an essential role in regulating tumor proliferation, invasion and migration (Li et al., 2022). In liver hepatocellular carcinoma (HCC), LINC00958 sponged miR3619-5p up-regulates the liver cancer-derived growth factor (HDGF) expression, which promotes HCC progression and adipogenesis. In addition, LIN00958 directly acts on NUDT19 and activates the mTORC1/P70S6K signaling pathway. NUDT19 overexpression and mTORC1 activator MYH1485 reversed the inhibitory effects of LINC00958 silencing on HCC proliferation, migration, and EMT process (Zuo et al., 2020; Lan et al., 2021). Jiang et al. (2021b) found that LINC00958 acts as an oncogenic lncRNA to regulate EC progression by modulating the miR-145-3p/TCF4 axis. A study by He et al. (2018) found that the interaction of LINC00958 and WDR5 increases the expression of VEGF-C and promotes LNM of bladder cancer. The molecular mechanisms in other cancers are shown in Table 5.

**TABLE 5:** Molecular mechanisms of LINC00958 oncogenesis in various cancers.

Cancer type	Expression	Molecular mechanisms	References
Bladder cancer	Upregulate	LINC00958 epigenetically upregulated VEGF-C expression by directly associating with WDR5, a core subunit of human H3K4 methyltransferase complexes	He et al. (2018)
Nasopharyngeal carcinoma	Upregulate	LINC00958 was found to serve as a molecular sponge of microRNA-625 (miR-625), thereby upregulating NUAK family SNF1-like kinase 1 (NUAK1) in NPC cells	Chen et al. (2019b)
Hepatocellular carcinoma	Upregulate	LINC00958 silencing inhibited the proliferation, migration, and EMT process of HCC *via* inhibiting NUDT19 mediated mTORC1/P70S6K signaling pathway	Lan et al. (2021)
Hepatocellular carcinoma	Upregulate	LINC00958 sponged miR3619-5p to upregulate hepatoma-derived growth factor (HDGF) expression, thereby facilitating HCC lipogenesis and progression. METTL3-mediated N6-methyladenosine modification led to LINC00958 upregulation through stabilizing its RNA transcript	Zuo et al. (2020)
Cervical cancer	Upregulate	LINC00958 could regulate RRM2 by competing to miR-5095, which regulates cell sensitivity to radiotherapy in cervical cancer	Zhao et al. (2019)
Osteosarcoma	Upregulate	LINC0095 promotes tumorigenesis and metastasis in osteosarcoma by competitively inhibiting miR-4306 expression, leading to elevated expression of CEMIP	Zhou and Mu (2021)
Glioma	Upregulate	LINC00958 acts as an oncogenic gene in the glioma genesis through miR-203-CDK2 regulation	Guo et al. (2018)
colorectal cancer	Upregulate	LINC00958 promoted MAPK1 expression and cell proliferation and suppressed cell apoptosis and radiosensitivity by targeting miR-422a	Liang et al. (2021)
Oral squamous cell carcinoma	Upregulate	cytoplasmic expression of LINC00958 in OSCC cells, and revealed that LINC00958 sequestered miR-627-5p to upregulate YBX2 expression	Chen et al. (2019a)
	Upregulate	The modulation of LINC00958 for the OSCC tumorigenesis through the miR-185-5p/YWHAZ axis	Wang et al. (2020)
Tongue squamous cell carcinoma	Upregulate	LINC00958 acted as a ceRNA by competitively sponging miR-211-5p	Jia et al. (2021)
Breast cancer	Upregulate	m6A methyltransferase-like 3 (METTL3) gave rise to the upregulation of LINC00958 by promoting its RNA transcript stability	Rong et al. (2021)
Head and neck squamous cell carcinoma	Upregulate	LINC00958 is a direct target of c-Myc and can enhance the transcriptional activity of c-Myc, thus to form a positive feedback gene network in HNSCC cells, and in turn to modulate HNSCC cell resistance to chemo- and radiotherapy	Huang et al. (2019)
Gastric cancer	Upregulate	methylated RNA immunoprecipitation sequencing (MeRIP-Seq) found that there were m6A-modificated sites in LINC00958, and moreover m6A methyltransferase KIAA1429 catalyzed the m6A modification on LINC00958 loci	Yang et al. (2021)
Endometrial cancer	Upregulate	LINC00958 acts as an oncogenic lncRNA to regulate EC progression by modulating the miR-145-3p/TCF4 axis	Jiang et al. (2021b)

In recent years, increasing studies have shown that TMB and MSI correlate with tumor-infiltrating lymphocytes and response to immune checkpoint inhibitors (ICIs) and can be predictive of response after immunotherapy (Samstein et al., 2019). Hence, we further explored the association of LINC00958 expression with TMB and MSI. The results revealed that LINC00958 expression was highly correlated with TMB in 12 tumor types and MSI in 7 tumor types. Additionally, patients with different TME characteristics play an important role in mediating late metastasis, immune escape and immunotherapy suppression (Wu et al., 2020). Our findings show that LINC00958 overexpression in a variety of tumors is negatively correlated with stromal and immune cell scores, suggesting that LINC00958 has an essential role in TME. These results demonstrated that LINC00958 had a non-negligible significance in tumor immunotherapy. However, the mechanism by which LINC00958 has a role in tumor immunotherapy remains to be further explored.

We further analyzed the mechanism of action of LINC00958 affecting tumor development using the MEM database and performing target gene prediction, GO analysis, and KEGG analysis. The results showed that the expression of S100A2, DKK3 and SCHIP1, which play a role in colorectal cancer (Bulk et al., 2009), HNSC (Katase et al., 2018), and gallbladder cancer (Gondkar et al., 2019), was significantly correlated with the expression of LINC00958. LINC00958 has been found to be associated with cell adhesion, extracellular matrix organization, and human papillomavirus infection in head and neck squamous cell carcinoma (Xiong et al., 2020b). Jiang et al. (2021a) found that LINC00958 induced a decrease in SIRT protein expression, further reducing P53 levels to influence oral squamous cell carcinoma progression. Our study also indicated that LINC00958 might affect tumorigenesis and progression through the P53 signaling pathway, extracellular matrix organization, and human papillomavirus infection.

There are several limitations to the study. Firstly, all of the included studies are from China. To solve this problem, we further verified our results by using a public database to improve the credibility of our conclusions. Secondly, the cutoff values for the high and low expression of lncRNA in all included studies are inconsistent, and some studies did the cut-off values that they used. Additionally, HRs and 95% CIs of some studies were extracted by KM curves, which may impact the accuracy of the outcomes.

## Conclusion

In summary, the overexpression of LINC00958 is significantly related to poor OS, positive LNM, advanced degree of infiltration, and advanced TNM stage in various cancers. LINC00958 might act as an oncogenic factor affecting tumor progression. With its ability to be used to discover potential therapeutic targets and improve cancer diagnosis and prognosis. Therefore, LINC00958 might serve as a potential prognostic biomarker and therapeutic target for a variety of cancers. However, high-quality research with larger sample size is still needed in the future to confirm our results. The oncogenic mechanism and related therapeutic targets of LINC00958 deserve to be further explored in the future.
